# Access to healthcare for long-term conditions in women involved in street-based prostitution: a qualitative study

**DOI:** 10.1186/s12875-015-0331-9

**Published:** 2015-09-03

**Authors:** Emma L. Mastrocola, Anna K. Taylor, Carolyn Chew-Graham

**Affiliations:** Health Education South West, Vantage Office Park, Old Gloucester Road, Hambrook, Avon, Bristol, BS16 1GW UK; Faculty of Health Sciences, University of Bristol, Senate House, Tyndall Avenue, Bristol, BS8 1TH UK; Research Institute, Primary Care and Health Sciences, Keele University, Keele, Staffordshire ST5 5BG UK

## Abstract

**Background:**

Women involved in street-based prostitution (SBP) have well-documented health problems specific to their occupation, but access to care for other chronic health problems has not been explored. Primary care is seen as the optimal context to deliver care for people with long-term conditions because it is accessible, efficient, and can tackle inequalities related to socioeconomic deprivation. We aimed to explore the perspectives of women involved in SBP about access to health care for their long-term conditions.

**Methods:**

This was a qualitative study with women accessing a third sector organization in North West England. Semi-structured interviews were conducted with sixteen women involved in SBP and accessing support. Data were analysed using the principles of constant comparison and a framework approach.

**Results:**

Women described how they were living with ill health, which they found difficult to manage, and often impacted on their work. Women reported poor access to care and viewed any ensuing consultations in primary care as unsatisfactory.

**Conclusion:**

This study highlights the unmet health needs of women who work in SBP, not just related to their occupation, but due to their co-morbid long-term conditions. Access to primary care was reported to be problematic and interactions with general practitioners not fulfilling their expectations, which impacted on future consultation behaviour. Understanding the health-seeking behaviours and self-management strategies of women involved in SBP with chronic health problems is essential in the design and commissioning of services and may reduce unscheduled care in this under-served group.

## Background

Long-term conditions (LTCs) are increasingly important determinants of quality of life and healthcare costs in populations worldwide [1]. Primary care is seen as the optimal context to deliver care for people with long-term conditions because it is accessible, efficient, and can tackle inequalities related to socioeconomic deprivation [2, 3]. However, access to primary care is known to be difficult for some groups of patients [4]. Women involved in street-based prostitution (SBP) are an under-served and under-researched group, and their health is a source of international concern [5]. These women are a high-risk population, and street-based workers are at greater risk than their parlour-based counterparts due to their increased prevalence of intravenous drug use and poorer engagement with healthcare [6]. Women have specific health needs relating to their occupation and lifestyle [7], being more likely to use drugs, experience occupational violence and have a less stable home environment [6, 8, 9]. Women involved in SBP often have complex socioeconomic backgrounds and chaotic lifestyles [7, 10], factors that may have led them into prostitution initially [11, 12]. Poor health may be a direct result of their lifestyle [13]; the standardised mortality ratio for those involved in prostitution in the United States is three times greater than that of the general population [14]. They commonly experience social exclusion and stigma related to their occupation [10, 15], and belonging to a marginalised group subject to socioeconomic disadvantages is in itself damaging to health [16–18].

**Table 1:** Patient demographics

Number of women interviewed	16
Age range	Mean 38.5 years
	Range 22–60 years
% White British	94 %
Length of time involved in SBP	Mean 16.6 years
	Range 4–40 years
Types of accommodation	Council, rented, women’s shelters, or homeless
% reporting partner	50 %
% reporting having had children	69 %

**Table 2:** Conditions disclosed

Participant number	Conditions disclosed (terminology used by participants)
1	Diabetes
2	Depression, anxiety, psychosis, asthma
3	Asthma, depression, anxiety, chronic back pain
4	Asthma, recurrent chest infections, CVA, recurrent alcohol-related seizures, depression
5	Depression, anxiety, chronic lower back pain
6	Aortic and tricuspid regurgitation (secondary to endocarditis), chronic DVT
7	Osteoarthritis, diabetes, hypertension, ‘stress’
8	Depression, ‘chest problems’
9	Chronic back pain, emphysema, rheumatoid arthritis, depression
10	Depression, ‘fits’, ‘heart problems’
11	Hepatitis C, psychosis, paranoia
12	Depression, chronic DVT, chronic lower back pain
13	Bipolar disorder, alcohol dependency
14	Depression, paranoia, asthma
15	Asthma, recurrent bronchitis
16	Depression, anxiety
Data extracts	
Data extract 1	[when blood sugars are high] *I just wait for it to come down a bit. If I eat something then it will go up and if I wait a bit, I take it again, see how much it’s gone down.* (P7)
Data extract 2	*When my breathing gets bad, I open the windows to get more air, erm and not do too much physical stuff*. (P3)
Data extract 3	*Well, it probably does, don’t it? Brings on panic attacks and everything. Because you make your nerves bad, you go out there and you don’t know who you’re gonna meet. You don’t know whether you’re gonna be killed or anything.* (P4)
Data extract 4	*If I get a punter and he’s in a wagon and it’s them steps… I’ve gotta take it easy getting into the wagon and getting out, I’ve gotta come down backwards. It’s easy to get out of the car, I just have to roll forwards. I just have to rock and get hold of something and sometimes when I do a punter they come round and help me out. Like if I get out of a taxi, I’ve gotta give him the crutches and then I grab hold of the handles so I can get up.* (P7)
Data extract 5	*I don’t worry about my health any more. I used to but not now… Cos there’s no use worrying.* (P1)
Data extract 6	*I’m unhealthy. I just take each day as it comes.* (P3)
Data extract 7	*Lack of time, money, lack of support, lack of a place to live even. If you’re sleeping here, there and everywhere, they’re not thinking about going to see the doctor…* (P14)
Data extract 8	*They’ve [GP surgery] changed their number and it’s a weird number and you can spend up to a fiver on your phone, when it goes to a main thing and then there. Basically now if I’ve got no credit on my phone, I just have to grin and bear whatever’s going on.* (P3)
Data extract 9	*I took myself to hospital, but actually that was like when I got a stay. Other times I’ve took meself to hospital when I’ve not been feeling well and they told me to just leave.* (P5)
Data extract 10	*I had pneumonia for four weeks. Just thought it was a bug. Couldn’t get off the settee. I was sweating and – I just got the ambulance.* (P6)
Data extract 11	*I went to the hospital… probably about three or four times this year… Overdoses and just one where I couldn’t think, I just couldn’t, I don’t know what was going on in me head everything was just all over the shop.* (P8: [describing recent overdoses])
Data extract 12	*I’ve had a few [GPs], moved from one place to another. For a few years I didn’t even bother going to see the GP.* (P11)
Data extract 13	*I went in to see the doctor and I had this full list, anti-depressants, drinking alcohol and whatever else, and I end up losing me temper in there. He just wanted to deal with one thing at a time. Told me to come back for the others. I lost me temper in there and I got a letter in the post saying I was no longer registered there.* (P8)
Data extract 14	*I wanted a female doctor because they listen and I feel I’m getting my needs met whereas with this particular doctor who I, sits back like that (puts arms behind her head) in his chair and erm it’s sort of like everything’s like a no and you’re being interrogated as to why you want this, why you want that and if he thinks you don’t need it, you won’t get it.* (P3)
Data extract 15	*It depends what it’s for. I had laser treatment [on her cervix] and that was a man and I didn’t like that at all. That’s why I never went back and I made sure I got a woman this time. Things like that I prefer a woman. But to actually talk to, I prefer a man. They’re more clinical men so they get to the root of things more easily I think.* (P14)
Data extract 16	*Well, some can be nice and some can be funny with ya. Cos you’re an alcoholic they think, oh don’t bother with her, you know what I mean. Think you’re a waste of time don’t they?* (P4)
Data extract 17	*Sometimes when you go in places and you say what do you do for an occupation and I sit there and think, should I tell em I’m a working girl or should I not? I weight up whether I should or not, whereas I should just be able to go in and say yeah I am, so what’s that gotta do with anything?* (P2)
Data extract 18	Int: *Why would you not tell them [your GP about your work]?*
	P14: *Couldn’t take the judgmental looks and you always get that. Even if they say no no I’m not judgmental, you always get the judgmental looks*. (P14)
Data extract 19	*[names support worker] Takes me to appointments, phones places up and sorts out everything that needs sorting out… I’ve known her a long time and when I was working on the street she used to bring me brews and everything, so she’s more of a mate than a worker so I can relate to her and I feel I can talk to her.* (P13)
Data extract 20	*One of the people that work in here [MASH] took me [to A + E] and she had to go in and basically say ‘Look, I work at MASH and she’s not right, she needs help.’ You know it took her to go in, not me…and she managed to get me in straight away and I was seen.* (P2)
Data extract 21	*It’s nice to know that you’ve got someone there that does listen to ya, not just in one ear and out the other, do ya know what I mean.* (P15)

Although ten years out of date, the most commonly used data estimates that there are 80,000 sex workers in the UK [19]. Estimates suggest that, of the 50–80,000 *female* sex workers, around 28 % work in street prostitution, while the remaining 72 % work in indoor establishments and as escorts.

Most UK research conducted into the health of women involved in SBP largely focuses on public health issues, women’s health and safety needs relating to their occupation, including substance use, sexual health and access to screening [4, 9, 10, 18–23]. There is a limited literature on mental ill health with offenders convicted for prostitution related offences. However, it has been shown that over 48 % experienced psychological problems or depression compared to 33 % of other offenders [24]. 68 % of women in prostitution meet the criteria for Post Traumatic Stress Disorder, in the same range as victims of torture and combat veterans undergoing treatment [25].

Access to care for physical health relating to LTCs in SBPs has not, however, been the focus of research [26], although evidence suggests that those involved in SBP may have numerous health problems [26, 27], and the prevalence of physical and psychological ill health and chronic disease is greater in this group than the general population [12, 13]. In one study, street-based women were shown to report significantly more chronic health problems than their parlour-based counterparts [6], indicating that women involved in prostitution are not a homogenous group in terms of health needs. Additionally, coordination of care for patients with LTCs is currently poor, and those suffering from a long-term condition have a lower quality of life [28], therefore these women are doubly disadvantaged.

Despite their high-risk status, patterns of accessing health services and receiving appropriate treatment are known to be poor [29], suggesting that street-based women experience significant barriers to accessing healthcare. They may not use mainstream services effectively and present late, if at all [6], resulting in higher rates of hospital attendance and admission compared to the general public [29]. Women may instead utilise third-sector organisations, which, while valuable for the management and support of problems associated with sexual health, may be unable to manage co-morbid physical health problems. In addition, access to such services may be opportunistic or haphazard, rather than providing the patient education and advice about medication required in supporting the management of long-term health problems.

Previous research into patterns of access to healthcare and experience of healthcare services for women involved in SBP has been conducted outside the UK, where models of healthcare, the legalities of prostitution, and the criminal justice system may differ from those in the UK [18, 30].

This study examines the perceptions and experiences of women involved in SBP regarding their ability to access care for long-term physical and mental health problems, and their interaction with healthcare services. Understanding the health-seeking behaviours and self-management strategies of female street-based workers with chronic health problems could help in designing and commissioning services, reduce unnecessary hospital admissions and improve patient education around self-management of chronic conditions in this under-served group.

## Method

This paper reports a qualitative study in which semi-structured interviews were conducted with street-based women involved in street-based prostitution. Ethical approval (reference number: *ethics/11355*) was granted by the University of Manchester Research Ethics Committee.

Potential participants were contacted through Manchester Action on Street Health (MASH)^1^. Inclusion criteria were women attending MASH, aged 18 or over, who were working in street-based prostitution and who had a long-term condition. Women were excluded from the study if they were younger than 18 years old or were unable to read or comprehend English. Women were recruited opportunistically to the study; women accessing the outreach or drop-in service between April and July 2012 were given a patient information leaflet and, if they expressed an interest in participating, were invited to have a brief discussion with the researcher (EM) and if they agreed, a time to be interviewed was arranged.

Interviews were conducted with written consent and were audio-recorded. Each interview lasted up to an hour and took place in a private room at MASH. Participants were reimbursed for their time (acknowledging that the woman might have been working during the time she was interviewed). Interviews continued until data saturation was reached. A topic guide, developed from the literature, explored women’s understanding of health and disease, ideas around prioritisation of health, self-management of their illness, help-seeking behaviours, challenges to accessing health services, and relationships with healthcare practitioners.

Interviews were transcribed verbatim and the recordings erased. Data analysis was inductive, taking a constant comparison approach [31] and testing emerging themes in subsequent interviews. Using an approach derived from framework analysis [32], data coded under each theme was summarised in tables, which enabled the identification of similarities and differences between transcripts, and to note deviant cases. Discussion amongst the authors led to agreement of themes.

## Results

### Participants

Sixteen women were interviewed, with a range of health problems. Three overarching themes will be presented: living with ill health, poor access to care, and unsatisfactory interactions with primary care. Illustrative quotes are provided, identifiable by participant number (Table 1 and Table 2).

### Living with ill health

Most respondents described multiple health problems, including mental and physical co-morbidities, and the impact of these on their work. Women described limited understanding of their conditions and few self-management strategies, which were barriers to them making informed health choices about managing their symptoms and seeking care.

<DE1>

<DE2>

Additionally, women described how their work contributed to psychological distress.

<DE3>

Some women also described how their physical symptoms made it difficult to cope with their work.

<DE4>

The narratives suggested a lack of agency, and fatalistic approach to health and illness.

<DE5>

<DE6>

Such attitudes impacted on the wish to seek care, but women then described multiple barriers when they had made a decision to seek help.

### Poor access to care

All participants talked about the challenges they face when trying to access services. Women described multiple barriers to accessing primary care. Those women who were homeless described how they had been unable to register with a general practice.

<DE7>

Even the telephone system at practices could be a barrier to women.

<DE8>

So, women described accessing A&E in preference to primary care, with some women reporting then perceived lack of care from A&E.

<DE9>

Many women described how they did not seek help when they first experienced symptoms, rather waiting until they felt seriously unwell and sought urgent care.

<DE10>

Women often reported help-seeking at a point of crisis, particularly for mental health problems.

<DE11>

Women’s prior experiences of help-seeking was reported to influence future decisions.

### Interactions with primary care

Women described a lack of continuity of care, some blaming their occupation and the need to move around and change practices.

<DE12>

Women reflected on their experiences of interacting with primary care practitioners. Many reported negative experiences of interactions, which led to them being unwilling to seek help again when feeling unwell. Some women expressed a preference for a female doctor for certain problems, but others preferred a male GP.

<DE13>

<DE14>

<DE15>

Women made judgments about the approachability of GPs, but also felt that GPs made judgements about them.

<DE16>

This fear of judgment also affected the decisions the majority of the women made around disclosing their profession to their GPs, with most saying they would never tell them. They also cited embarrassment, a need for privacy and not seeing the relevance of disclosure to their healthcare needs, as reasons not to tell their GPs that they worked in prostitution.

<DE17>

<DE18>

Women reported that the advocacy role of MASH made it easier for them to access healthcare as they could be guided by the support worker in how best to seek help.

<DE19>

<DE20>

All women valued this advocacy role, which helped them negotiate what seemed to be a complex and unwelcoming healthcare system. One woman reported a positive relationship with her GP, but this may be regarded as a deviant case.

<DE21>

## Discussion

### Summary of main findings

Most women interviewed disclosed more than one health problem, often a combination of physical and psychological, in addition to major social difficulties. Women’s knowledge of their physical conditions and how to optimally manage them seemed poor. Their chaotic lifestyle meant that their perceptions of the seriousness of their health problems were poor, resulting in help-seeking at a time of crisis. Their help-seeking, however, was also influenced recursively [33] by previous negative experiences at a number of different levels. Access to primary care was described as difficult both in the systems (the need for an address, the telephone system) and in personal interactions with GPs, with women feeling both that the ten minute consultation, did not allow for the discussion of multiple problems, and that they were judged by the GP. Women described the valuable support obtained from MASH support workers, who acted as advocates in help-seeking for their physical and mental health needs. Such workers could also help the women navigate a complex health care system.

### Strengths and limitations

This is the first study to explore the perspectives of women involved in SBP about impact and management of their chronic health problems, help-seeking and access to care in the UK. Using qualitative methods with semi-structured interviews enabled participants to discuss issues and experiences that were important to them, and talk freely about their concerns regarding their health.

Whilst contacting women involved in SBP through MASH enabled access to this vulnerable group of women, it may be that we did not speak with women who were most marginalised and vulnerable, with no formal care or support. It is likely that their problems would be greater than those described by our participants.

This study was conducted in one third-sector organisation in one city, and, whilst results cannot be generalised across the UK, it is likely that they paint a picture of un-met need and poor access to care for this patient group. Most participants (94 %) were White British, thus the needs of women from other ethnic groups have not been explored.

### Comparison with existing literature

The difficulties in accessing primary care faced by women in SBP has been reported to be due to a variety of factors, including lack of knowledge of available services [10, 34], and practical challenges such as lack of transport, or inability to afford public transport, and difficulty attending appointments during practice opening times [11, 18]. Our study supports previous literature where the fear of being judged by other patients or by health workers upon disclosure of occupation [11, 15, 18, 27, 29] was a factor in limiting seeking help. Using Dixon-Wood’s framework of candidacy for healthcare [35], we have demonstrated that our respondents were unclear over what made them ‘candidates’ for care for their physical health problems, and from which service to seek help. Recursivity was seen in womens’ accounts of how they chose between healthcare services, particularly in the choice to use A&E in preference to primary care [33]. Similar tensions are seen in accounts of other vulnerable groups such as people with mental health problems.

‘Permeability’ offers a way to conceptualise the impact of these barriers [35]. Highly permeable services require less work and fewer resources from patients who access them - for example, A&E in the UK which is open at all times. A service that seems accessible may in fact be impermeable to particular patient groups. For example, despite general practices being locally available, with designated systems for urgent access, women in our study described that they were, in fact, impermeable because of factors such as (in)ability to register due to being homeless, complex and costly telephone systems, and travel costs. In addition, powerful accounts of previous negative encounters with primary care professionals also recursively influence future help-seeking.

### Implications for practice

Women involved in SBP described multiple mental and physical health and social needs, and describe barriers encountered in attempting to access to health-care. They thus constitute an important under-served group [7, 36].

The emphasis of policy, and education and training of GPs and their staff, should be on shaping patient-practitioner interactions within which candidacy for healthcare use is recursively established, and on intervening in the experiences of services, as these frame patients’ future healthcare choices. Women struggled with accessibility, so a more flexible service should be offered to enable them to more easily access care. Additionally, more education should be provided to GPs in helping them to communicate with women and better recognize their needs.

The concealed nature of the industry [37] makes commissioning services specific to this patient group challenging, and women may not wish to access a specific service, which would mean having to disclose the nature of their work to the health care practitioner. However, primary care does not currently meet the needs of these women.

The important role played by support workers in directing help-seeking could be harnessed by commissioners, together with ensuring that primary care is aware of such groups, and responsive to requests for help. In addition, commissioners need to ensure longer-term contracts for such third sector services, to enable a sustainable service to be available consistently to support women involved in prostitution navigate the health care system and learn to manage their long-term physical and mental health problems [28].

## Conclusion

Primary care is seen as the optimal context to deliver care for people with long-term conditions; access to primary care is known to be difficult for some groups of patients, including women involved in SBP. Women have specific health needs relating to their occupation and lifestyle, and are living with chronic health problems which impact on their work. This study suggests that previous unsatisfactory primary care encounters and the fear of being judged by health care practitioners impact on willingness to seek help. Understanding the health-seeking behaviours and self-management strategies of women involved in SBP with chronic health problems is essential in the design and commissioning of services and may reduce unscheduled care in this under-served group.

## Abbreviations

A&E: Accident and emergency
CVA: Cardiovascular accident
DVT: Deep vein thrombosis
LTCs: Long-term conditions
SBP: Street-based prostitution

## Footnotes

1 MASH is a charity which provides free and confidential information and support for women involved in prostitution in Greater Manchester, England.
